# Editorial: Sequencing and phylogenetic analysis as a tool in molecular epidemiology of veterinary infectious diseases

**DOI:** 10.3389/fvets.2023.1236155

**Published:** 2023-07-13

**Authors:** Christina M. Leyson, Moh A. Alkhamis, Iryna V. Goraichuk

**Affiliations:** ^1^Balik Scientist Program, Department of Science and Technology, Taguig, Philippines; ^2^Department of Epidemiology and Biostatistics, College of Public Health, Health Sciences Center, Kuwait University, Kuwait City, Kuwait; ^3^Exotic and Emerging Avian Viral Disease Research Unit, Southeast Poultry Research Laboratory, U.S. National Poultry Research Center, Agricultural Research Service, United States Department of Agriculture, Athens, GA, United States; ^4^National Scientific Center Institute of Experimental and Clinical Veterinary Medicine, Kharkiv, Ukraine

**Keywords:** genome sequencing, next-generation sequencing, sanger sequencing, phylogenetic analysis, metagenomics, molecular surveillance, molecular epidemiology

In the past few decades, the rapid development and decreasing costs of sequencing technologies dramatically changed the landscape of epidemiological studies and surveillance of infectious diseases. Subsequently, pathogen genomic studies have become the forefront tool for investigating emerging infectious disease epidemics and supporting decision-making processes related to the mobilization of intervention resources. Since the introduction of next-generation sequencing (NGS), which expanded the capacity for whole genome sequencing (WGS), and the revolutionary growth of computational resources, viral or bacterial complete genomes can now be sequenced and characterized within a few days or even hours. Therefore, high-throughput sequencing technologies resulted in the exponential growth of genomic databases, unveiling novel insights into the biology, pathophysiology, and molecular epidemiology of infectious disease pathogens. Such significant developments in genome sequence technologies also resulted in important advances in the field of phylogenetic analysis. Modern analytical methods in phylogenetics improved the tracking and understanding of pathogen transmission and evolution of human and animal diseases. Thus, phylogenetic analysis of big genomic databases can be used to clarify key questions related to infectious disease epidemiology, such as the initial detection and characterization of outbreaks, accurate tracing of transmission chains between hosts, and dispersal among and within geographical regions. The threat of emerging and re-emerging infectious diseases continues to be a challenge to global public and animal health, in which sequencing is not just a critical tool in surveillance but also has a major role in a pandemic or outbreak response. The main objective of this Research Topic is to explore the current status and future perspectives of sequencing technologies in the control and prevention of infectious diseases, including the elucidation of diagnosis, molecular evolution, and epidemiology of infectious disease pathogens.

NGS has become a primary diagnostic and characterization tool for genomic surveillance of pathogens that threaten biosecurity and food safety. Kariithi et al., Kim H.-J. et al., and Abbas et al. demonstrated multiple approaches in utilizing sequencing and phylogenetic analysis resources for improving genomic surveillance of avian infectious bronchitis virus in poultry by characterization of phylogenetic relationships, detection of critical recombination events, assessing vaccination effectiveness, and identifying evolutionary origins of endemic and emerging strains. Furthermore, Kim S.-W. et al. were able to confirm that avian reoviruses circulating in poultry flocks were originating from wild birds using straightforward traditional molecular characterization tools, similar to what Goraichuk et al., did with the Newcastle disease virus. However, Baek et al. went a step further by implementing Bayesian phylodynamic analysis to shed deeper insights into the evolutionary epidemiology of H5N8 avian influenza viruses. They demonstrated that wild birds were the ancestral host for multiple introductions of H5N8 viruses into poultry, but domesticated ducks more important in virus circulation and transmission among poultry flocks.

African swine fever continues to be the most important devastating pathogen to swine populations, causing unprecedented annual economic loss on a global scale. NGS and WGS also continue to be the most essential tools for providing critical genetic, epidemiologic, therapeutic, and vaccine development resources for African swine fever intervention efforts, as illustrated by Hyeon, Tseren-Ochir, et al., Gallardo et al., and Kim G. et al. Furthermore, the combination of WGS and phylodynamic analytical approaches was used by Pamornchainavakul et al. to unveil novel findings on how the rapid recombination events among porcine reproductive and respiratory syndrome virus 2 strains can accelerate their genetic mutations leading to the emergence of more virulent strains. Similarly, Wei et al. were able to identify evolutionary characteristics and geographical origins of the lumpy skin disease virus in cattle, using multiple gene segments of a strain isolated from an outbreak in China in 2009. Rossi et al. extended their genomic analytical pipeline by integrating ecological niche models to quantify the role of environmental and demographic risk factors in shaping the evolutionary epidemiology of *Mycobacterium bovis* in Cameroon, which is considered a novel and critical step in improving genomic surveillance of infectious diseases.

Genomic surveillance of infectious diseases in wildlife is the foundational pillar of implementing the One Health concept globally. Indeed, the rapid emergence and spread of West Nile and Rabies viruses from wild animal origins played an important role in building the foundation of the One Health concept, which necessitates integrating disease surveillance of humans, wildlife, and domestic animals. In this Research Topic, Chung et al. and Hyeon, Helal, et al. used NGS and WGS approaches to deeply characterize the genetic features of West Nile virus and lyssaviruses from wild animals in selected regions in the United States in order to shed important insights about their origins and transmission dynamics. Additionally, it is important to include genomic surveillance of pathogens in exotic pet animals, such as reptiles, to monitor evolutionary characteristics that may result in the emergence of novel pathogens, as demonstrated by Varga-Kugler et al..

Continuous development and revision of NGS and WGS pipelines are critical for ensuring the sustainable generation and accumulation of sound and reliable genetic data. In this Research Topic, Lorente-Leal et al. revised the performance and agreement of four commonly used pipelines for WGS data analysis of *M. bovis*, the causative agent of bovine tuberculosis, Butt et al. highlighted the feasibility and utility of long-read random sequencing approaches to identify pathogens in clinical samples, and Cho et al. developed a novel tiling amplicon PCR method for feasible and rapid sequencing of complete genomes in clinical samples.

Our Research Topic reinforces the importance of sequencing technologies in changing the landscape of infectious disease surveillance in animals and humans, with the aim of highlighting NGS and WGS approaches that continue to become more feasible and accessible globally. This Research Topic highlights the versatility of techniques for bench science and sequence analysis in its application to many problems in infectious diseases of veterinary importance. In this Research Topic alone, investigations on 13 infectious diseases of poultry, swine, bovine, and wildlife were presented. Furthermore, the authors of these investigations span 23 countries and five continents, highlighting the accessibility of NGS techniques and its continued lowering of costs. In this Research Topic, we aspire to further motivate physicians, veterinarians, epidemiologists, microbiologists, diagnosticians, and other scientists from related fields to make sequencing technologies the gold standard for the diagnosis and surveillance of infectious disease pathogens in order to improve their current and future intervention efforts.

## Author contributions

All authors were involved in the writing of this editorial and editing contributions to this Research Topic.

